# Editorial: Understanding Diabetic Foot Disease: Current Status and Emerging Treatment Approaches

**DOI:** 10.3389/fendo.2021.753181

**Published:** 2021-09-16

**Authors:** Nikolaos Tentolouris, Michael E. Edmonds, Edward B. Jude, Prashanth R. J. Vas, Chris A. Manu, Anastasios Tentolouris, Ioanna Eleftheriadou

**Affiliations:** ^1^First Department of Propaedeutic and Internal Medicine, Medical School, National and Kapodistrian University of Athens, Laiko General Hospital, Athens, Greece; ^2^Diabetic Foot Clinic, Kind’s College Hospital National Health Service. (NHS) Foundation Trust, London, United Kingdom; ^3^Department of Endocrinology, Tameside Hospital NHS Foundation Trust, Manchester, United Kingdom

**Keywords:** diabetic foot ulcers, wound healing, diabetic neuropathy, charcot foot, vitamin D

Diabetic foot disease is a common and devastating complication of diabetes mellitus. Diabetic foot ulceration has an annual incidence of 2.2%, while the lifetime risk of developing foot ulceration among people with diabetes reaches up to 34% (1). Unfortunately, even after successful wound healing, the recurrence rates of diabetic foot ulcers (DFU) reach up to 40% within a year and it has been suggested to consider epithelialized DFU as being in remission rather than being healed (1). It is estimated that DFU, usually in association with infection or ischemia, precede around 75-85% of all diabetes-related lower limb amputations and remain the worldwide leading factor for non-traumatic lower extremity amputations (2). Moreover, DFU severity seems to be a more significant predictor of subsequent mortality than coronary arterial disease, stroke or peripheral arterial disease (3) and foot ulceration is associated with a 5-year survival of 50 to 60% after presentation (4).

Diabetic foot ulcers are commonly caused by minor or repetitive trauma on an area of the foot in a person with peripheral neuropathy and/or peripheral arterial disease (1). Foot deformities, previous history of DFU or amputation, Charcot osteoarthropathy and reduced joint mobility leading to altered biomechanical loading are other major risk factors of foot ulceration (1). Nevertheless, impaired wound healing is considered the main factor the leads to the development of chronic diabetic wounds (5). The pathophysiology of diabetic wound healing is complex, multifaceted, and not completely understood so far. Moreover, more than half of DFU become infected, which in turn further slows wound healing and increases the risk of foot amputation (1).

The manuscripts in this Research Topic describe several aspects of diabetic foot disease from the pathophysiological point of view, the importance of nutritional deficiencies, the early identification of the foot at risk, all through the association of diabetic foot syndrome with other diabetic complications.

Berlanga-Acosta et al. thoroughly reviewed current knowledge regarding the association of cellular senescence with diabetic wound healing impairment. The authors outline the main pathways of hyperglycemia-induced cellular senescence, such as mitochondrial dysfunction, endoplasmic reticulum, and oxidative stress that result in DNA chemical damage and further molecular responses that regulate cellular senescence. Moreover, they suggest that DFU healing impairment and chronicity are to a great extent pathogenetically mediated by the presence of senescent cells within the ulcer. There is a senescence-associated secretory phenotype that further exacerbates tissue inflammation and affects all wound healing involving cells, such as fibroblasts, keratinocytes, epithelial cells, and mesenchymal stem cells.

Vitamin D deficiency is associated with impaired insulin synthesis and progression of type 2 diabetes (6). Xiao et al. examined the association of vitamin D deficiency with diabetes complications - diabetic retinopathy, diabetic kidney disease and DFU - in a large cross-sectional study of 4,284 hospitalized Chinese individuals with type 2 diabetes. Vitamin D deficiency was common and more than 70% of the study population had low vitamin D levels. Vitamin D deficiency was significantly and independently associated with DFU prevalence, while a similar association was not observed with retinopathy and kidney disease. Moreover, a greater proportion of patients with DFU had vitamin D deficiency when compared with those without DFU. The authors suggested that assessing vitamin D status among patients with DFU may represent a modifiable risk factor and vitamin D supplementation may help in the prevention of diabetic foot ulceration.

Panagoulias et al. conducted a prospective study of 308 individuals with diabetes without a history of DFU or critical limb ischemia and evaluated whether the visual indicator plaster method (Neuropad^®^) could reliably predict foot ulceration within a 6-year follow-up period. The study demonstrated that foot skin dryness as assessed by the indicator plaster method predicted DFU development and had high sensitivity, while other neurological modalities used in the assessment of chronic peripheral somatosensory polyneuropathy, such as vibration perception threshold and neuropathy disability score, had high specificity for the identification of foot at risk. The authors emphasized that the indicator plaster method can be performed by the patient at home and its implementation in every day clinical practice may save time for health care professionals and improve clinical outcomes in individuals with diabetes.

Trindale et al. performed a detailed and comprehensive cross-sectional study investigating the association between ocular disease and peripheral or cardiac autonomic neuropathy among patients with type 2 diabetes and Charcot arthropathy, patients with type 2 diabetes without Charcot and healthy individuals. Patients with diabetes had more abnormalities in their ocular surface indicative of dry eye syndrome and ocular surface dysfunction when compared with healthy participants, while ocular parameters were most abnormal among participants with Charcot arthropathy. Moreover, several ocular parameters were associated with peripheral neuropathy and cardiac autonomic neuropathy in the diabetic cohort. The authors highlighted that patients with Charcot arthropathy experience severe neuropathy not only in the foot but also in all parts of their body, while dry eye disease symptoms and findings may be used as supplementary clinical tools for the assessment and monitoring of diabetic neuropathy.

The management of diabetic foot disease is a challenge for every health care provider. Unquestionably, further research is warranted to understand the underlying mechanisms and to fill the gaps of knowledge that would ultimately lead to successful management. Berlanga-Acosta et al. propose future therapeutic strategies addressed to control cellular senescence in diabetic wounds, while Xiao et al. suggest investigating vitamin D screening and/or supplementation as an ulcer prevention approach among people with diabetes. Early identification of the foot at risk in order to prevent DFU and most importantly by the patient themselves is highlighted by Panagoulias et al., whereas Trindale et al. suggest an alternative way of monitoring diabetic neuropathy and its complications. Each topic discussed in this issue provides a new insight into the approach of diabetic foot disease and could lead to further high quality research.

## Author Contributions

All authors contributed to the article and approved the submitted version.

## Conflict of Interest

The authors declare that the research was conducted in the absence of any commercial or financial relationships that could be construed as a potential conflict of interest.

## Publisher’s Note

All claims expressed in this article are solely those of the authors and do not necessarily represent those of their affiliated organizations, or those of the publisher, the editors and the reviewers. Any product that may be evaluated in this article, or claim that may be made by its manufacturer, is not guaranteed or endorsed by the publisher.

 
